# Are osteoporotic fractures being adequately investigated?: A questionnaire of GP & orthopaedic surgeons

**DOI:** 10.1186/1471-2296-7-7

**Published:** 2006-02-07

**Authors:** George Chami, Lee Jeys, Mathew Freudmann, Louise Connor, Mashood Siddiqi

**Affiliations:** 1 Department of Computer Science, Hull University, Hull, UK; 2 Yorkshire Higher Orthopaedic Training Rotation, Leeds, Yorkshire, UK; 3 West Midlands Higher Orthopaedic Training Rotation, Birmingham, West Midlands, UK; 4 Grange Group Practice, Fartown, Huddersfield, West Yorkshire, UK; 5 Metabolic Bone Unit, University Hospital Aintree, Longmore Lane, Liverpool, UK

## Abstract

**Background:**

To investigate the current practice of Orthopaedic Surgeons & General Practitioners (GP) when presented with patients who have a fracture, with possible underlying Osteoporosis.

**Methods:**

Questionnaires were sent to 140 GPs and 140 Orthopaedic Surgeons. The participants were asked their routine clinical practice with regard to investigation of underlying osteoporosis in 3 clinical scenarios.

55 year old lady with a low trauma Colles fracture

60 year old lady with a vertebral wedge fracture

70 year old lady with a low trauma neck of femur fracture.

**Results:**

Most doctors agreed that patients over 50 years old with low trauma fractures required investigation for osteoporosis, however, most surgeons (56%, n = 66) would discharge patients with low trauma Colles fracture without requesting or initiating investigation for osteoporosis. Most GPs (67%, n = 76) would not investigate a similar patient for osteoporosis, unless prompted by the Orthopaedic Surgeon or patient.

More surgeons (71%, n= 83) and GPs (64%, n = 72) would initiate investigations for osteoporosis in a vertebral wedge fracture, but few surgeons (35%, n = 23) would investigate a neck of femur fracture patient after orthopaedic treatment.

**Conclusion:**

Most doctors know that fragility fractures in patients over 50 years old require investigation for Osteoporosis; however, a large population of patients with osteoporotic fractures are not being given the advantages of secondary prevention.

## Background

History of a fragility fracture (defined as a fracture sustained following a fall from a standing height) is one of the recognised risk factors of osteoporosis and subsequent fractures [1]. The most common fractures associated with osteoporosis are fractures of hip, vertebrae and distal radius. It has been shown that a low trauma distal forearm fracture in a patient over 50 years old has a significant risk of osteoporosis with increased risk of further fractures [2-4] Women with vertebral fractures are 12 times more likely to suffer new vertebral fracture and twice as likely to experience hip fracture as women without vertebral fractures [5] Hip fractures are recurrent in 14% of women and Colles' fracture in 10%. Indeed, any post menopausal fracture is an independent risk factor for a future hip fracture.

The widespread screening for osteoporosis is not economically viable; therefore, screening should be targeted at high-risk populations. These include patients with low trauma fractures, women undergoing hysterectomy +/- oopherectomy and patients on long-term corticosteroids.

Recent pharmacological advances have improved the treatment of osteoporosis and may reduce the risk of subsequent fracture by up to 50% [6] The aim of the study was to find out whether patients with low trauma fractures are being investigated for osteoporosis either by the Orthopaedic Surgeons or GPs.

## Methods

A postal questionnaire was sent to 140 Orthopaedic Surgeons (Consultants and Specialist Registrars) in 20 hospitals in North West England. Clinical scenarios were given of women presenting to fracture clinic with the three common osteoporotic fractures. These were a 55 year old patient with a distal radial (Colles' fracture), a 60 year old patient with a vertebral wedge fracture and a 70 year old patient with a fracture neck of femur. The surgeons were asked whether or how they would routinely investigate these patients for osteoporosis. They were also asked whether they thought an Osteoporosis Nurse Specialist would provide a beneficial service.

A separate postal questionnaire was sent to 140 GPs in North West England matched in numbers and area to the 20 hospitals in the previous questionnaire. Demographic details were taken regarding the usual orthopaedic service used by the practice. The GP was asked to assume they had received a discharge letter from the Orthopaedic Surgeons regarding the same clinical scenarios as above, they were asked whether, or how they would investigate the patient for osteoporosis. They were also asked; who they felt should take the prime role in initiating investigations for osteoporosis and whether they thought an Osteoporosis Nurse Specialist would provide a beneficial service.

## Results

The response rate was 84% (n = 117) from orthopaedic surgeons and 81% (n = 113) from GPs. One questionnaire had to be excluded from a GP due to multiple responses to each question.

It was generally recognised [81% (n = 95) of Orthopaedic Surgeons & 96% (n = 108) of GPs], that low trauma fractures in patients over 50 years old required investigation for osteoporosis. There was also general agreement that an Osteoporosis Nurse may be beneficial [81% (n = 95) of Orthopaedic Surgeons & 94% (n = 106) of GPs]

**Figure 1:**
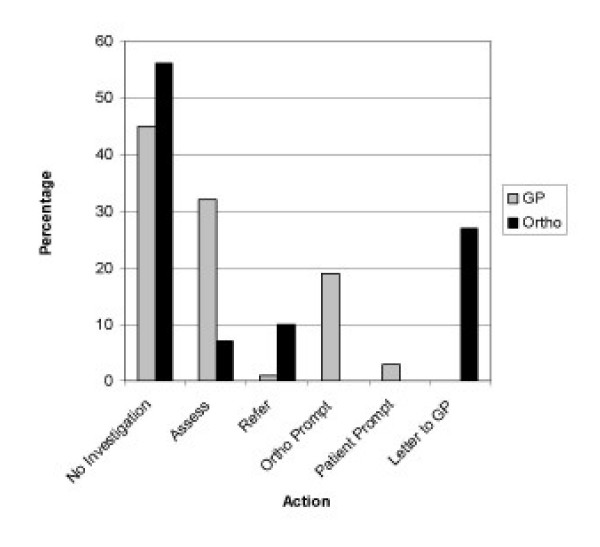
**Clinical scenario of low trauma Colles fracture**. The routine practice of GP's & Orthopaedic Surgeons with regard to investigation of underlying Osteoporosis in a 55 year old lady with a low trauma Colles fracture.

Orthopaedic surgeons responding to the clinical scenario of a 55 year old lady presenting with a low trauma Colles' Fracture, 56% (n = 66) would routinely discharge the patient with clinical details but not request investigation of underlying osteoporosis. However, 27% (n = 31) of surgeons stated that they would discharge the patient with a letter to their GP requesting further investigation into osteoporosis. Only 7% (n = 8) routinely assessed and/or started treatment for osteoporosis, whereas 10% (n = 12) routinely would refer a local osteoporosis clinic (Table 1, Figure 1).

**Table 1:** Numbers and Percentage response from GP's and Orthopaedic Surgeons to the three clinical scenarios.

ACTION	**55 Yr Old Colles #**		**60 Yr Old Wedge#**		**70 Yr Old NOF #**	
	
	**Ortho**	**GP**	**Ortho**	**GP**	**Ortho**	**GP**
No Request for Investigation	66 (56%)	51 (45%)	34 (29%)	28 (25%)	78 (66%)	46 (40%)
Letter to GP	31 (27%)	-	51 (43%)	-	19 (16%)	-
Ix if Prompted by Ortho	-	21 (19%)	-	9 (8%)	-	21 (19%)
Ix if Prompted by Patient	-	3 (3%)	-	3 (3%)	-	2 (2%)
Assess Patient/Start Treatment	8 (7%)	36 (32%)	16 (14%)	65 (58%)	13 (11%)	42 (37%)
Refer to Local Osteoporosis Clinic	12 (10%)	1 (1%)	16 (14%)	7 (6%)	7 (6%)	1 (1%)

**Figure 2:**
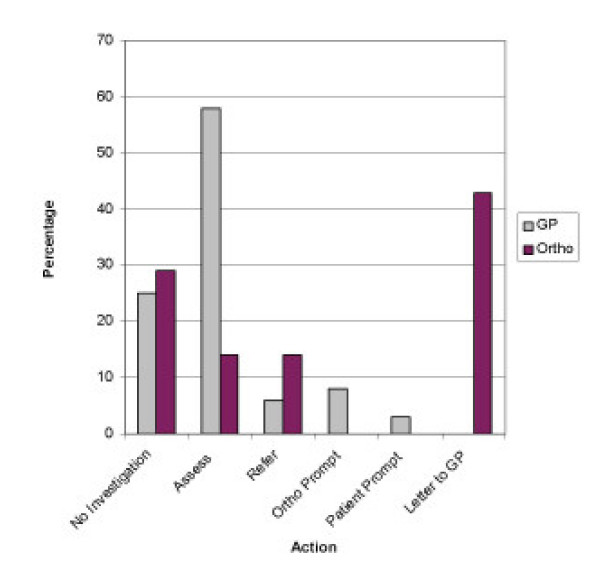
**Clinical scenario of low trauma vertebral wedge fracture**. The routine practice of GP's & Orthopaedic Surgeons with regard to investigation of underlying Osteoporosis in a 55 year old lady with a low trauma vertebral wedge fracture.

With regard to the vertebral wedge fracture, 43% (n = 51) of surgeons would routinely ask the GP to investigate for osteoporosis, but 29% (n = 34) would simply discharge the patient. However, 14% (n = 16) would routinely assess and/or start treatment for osteoporosis, while 14% (n = 16) would refer to a local Osteoporosis clinic (Figure 2).

**Figure 3:**
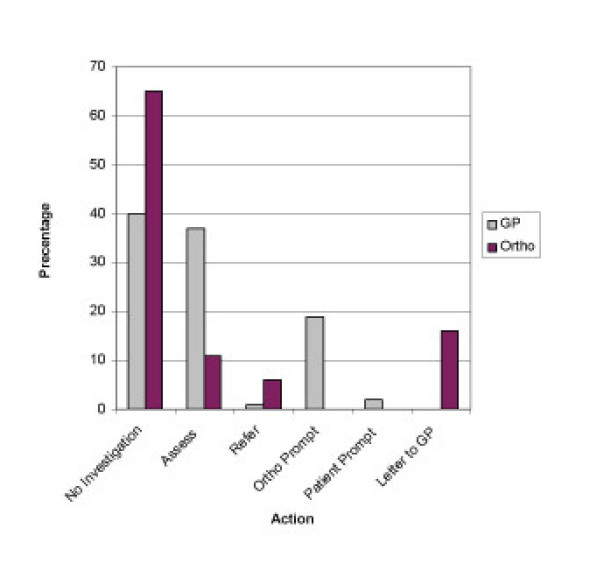
**Clinical scenario of low trauma fracture neck of femur**. The routine practice of GP's & Orthopaedic Surgeons with regard to investigation of underlying Osteoporosis in a 55 year old lady with a low trauma fracture neck of femur.

When faced with a neck of femur fracture, 66% (n = 78) would simply discharge the patient after orthopaedic treatment, 16% (n = 19) would ask the GP to investigate for osteoporosis. Although 11% (n = 13) would routinely assess the patient for Osteoporosis, 6% (n = 7) would refer the patient to the local osteoporosis clinic (Figure 3).

In response to the clinical scenario with a Colles Fracture, 45% (n = 51) of GPs felt they would routinely file the discharge letter assuming the Orthopaedic Service would have investigated the patient for Osteoporosis if indicated. A further 19% (n = 21) felt they would investigate the patient for Osteoporosis only if prompted by the Orthopaedic Surgeons, and another 3% (n = 3) if prompted by the patient. Only 32% (n = 36) would routinely assess the patient and/or start treatment for Osteoporosis, while 1% (n = 1) would refer to the local Osteoporosis clinic.

With regard to a Vertebral Wedge Fracture, 58% (n = 65) of GPs would routinely assess and/or start treatment for Osteoporosis, however, 25% (n = 28) would simply file the letter. A further 8% (n = 9) of GPs would investigate the patient if prompted by the Orthopaedic Surgeons, and 3% (n = 3) if prompted by the patient. Only 6% (n = 7) would routinely refer the patient to a local Osteoporosis clinic.

In the final clinical scenario of a Neck of Femur Fracture, 37% (n = 42) of GPs would routinely assess and/or start treatment for Osteoporosis, however, 40% (n = 45) would simply file the letter. A further 19% (n = 21) of GPs would investigate the patient if prompted by the Orthopaedic Surgeons, and 2% (n = 2) if prompted by the patient. Only 1% (n = 1) would routinely refer the patient to a local Osteoporosis clinic.

## Discussion

There over 200, 000 osteoporotic fractures each year in the UK, costing the NHS £942 million pounds [1]. Unfortunately, 1 in 3 women, and 1 in 12 men will suffer an osteoporotic fracture in their lifetime, however, the investigation and treatment of Osteoporosis is only cost effective in high risk populations. The consequences of these fractures can be disastrous; 50% of patients sustaining fractured neck of femurs will lose the ability to live independently and up to 33% will die within 1 year [1,7].

One such high risk population for osteoporosis, are those patients presenting with low trauma fractures. As this survey shows, both Orthopaedic Surgeons and GPs agree that patients with low trauma fractures over the age of 50 years require investigation for Osteoporosis. However, neither group in our study regularly investigates these patients, especially with the Colles' and Neck of Femur fractures populations. Orthopaedic surgeons are regularly in contact with this population, it would be logical that they either investigated or highlighted this population to the GP or local Metabolic Bone Physician for further investigation.

Although there have been several articles in the press dating back to 1998, emphasizing that a change in perspective and more holistic approach is required by Orthopaedic Surgeons with regard to Osteoporosis [8-10], attitudes appear to have changed little, according to the result of this study. In a survey of patients Pal [11] found only 15% of patients with fragility fractures subsequently received Bisphosphonates in 1999. In our survey only 17% of Orthopaedic surgeons would initiate treatment or refer a 55 year old lady with a low trauma wrist fracture to the appropriate service; a poor reflection of 6 years of osteoporosis education. Our study shows that it is an on-going issue, which has yet to be resolved; although there is wider awareness of secondary prevention for vertebral fractures, wide scale implementation of secondary prevention policies are yet to be established in many orthopaedic units. General practitioners need to be aware of these deficiencies in the current system and be pro-active in investigating patients with fragility fractures; Orthopaedic surgeons should at the very least highlight this subset of patients to the general practitioners or metabolic bone physicians. However, recent studies conducted in USA [12] and Canada [13] have shown this problem is not confined to the UK.

There is good evidence showing that low trauma distal radial fractures are a predictor of osteoporosis, associated with an overall 50% increase in risk of subsequent hip fracture and that there is a 2.3 fold increase in risk of hip fracture following Vertebral wedge fracture. Recent advances in the treatment of Osteoporosis mean that a 10% increase in average bone density would result in a 50% reduction in fractures [1]

There is also evidence that treatment of osteoporosis with bisphosphates, such as Aledronate and Risedronate can significantly reduce the risk of subsequent hip fractures. Studies have shown that treatment with alendronate can produce a 47% risk reduction for nonvertebral fracture [14], and treatment with risedronate can reduced the hip fracture by 41% (from 3.2% to 1.9%) when compared with placebo [15]. A trial by Meurier [16] showed that adding Calcium and Vitamin D supplements systematically to institutionalised elderly people reduced hip fractures by 23%. Some professionals suggest that it is advisable to initiate preventive treatment in all fragility fractures over the age of fifty, whatever the result of bone densitometry, feeling that it is never to late to try to prevent further fractures in high risk patients.

The results from the survey show that Vertebral Wedge fractures are relatively well treated by both GP's & Orthopaedic Surgeons. This may be because the link between these fractures and Osteoporosis has been well recognised recently. Both specialities may not appreciate the link between Colles fracture and subsequent Hip fractures.

The results show that, if Orthopaedic Surgeons requested that the GP investigate the patient, or informed the patient that they were at risk from Osteoporosis, then it would be possible to increase the amount of patients being investigated by GP's from 33% to 65% for distal radial fractures. If the profile of secondary prevention of Osteoporosis was increased through the medical press then it may be possible to increase this further.

The role of Osteoporosis Nurse Specialists in the identification of high risk populations is uncertain, however, the benefits of a person able to recruit patients over 50 years old with low trauma fractures to the Osteoporosis Clinic from Fracture clinic population would be obvious. There was general agreement by both GP's and Orthopaedic surgeons that they may provide a beneficial service, though there were questions raised regarding funding for such posts and subsequent pressure on Osteoporosis service. Several GP's commented on the lack of a local Osteoporosis service or access to Dexa Bone Density Scans, which is clearly not acceptable if osteoporosis is to be treated effectively.

In the forthcoming decades, it is predicted that there will be large changes in the demographics of the UK population, with the population size increasing from 59.2 million in 1998 to an estimated population 63.5 million in 2021. Also the mean age of the population will increase, as in 1998 the proportion of children was 13% greater than the proportion of pensioners, however, by 2008 it is predicted that the proportion of pensioners will overtake that of children [17]. Given these facts and combined with the increasing life expectancy, increasing health expectations and possibly tighter economic constraints on the NHS, the future trauma workload of Orthopaedic Surgeons will increase dramatically, unless steps are taken to prevent the tide of future osteoporotic fractures. This prevention has to take place many years before the benefits will be seen, and through the treatment of fragility fractures in the middle aged population now, it may be possible to reduce the numbers of subsequent fractures and silent epidemic of osteoporosis.

## Conclusion

There is awareness that low trauma fractures in patients over 50 years are associated with Osteoporosis, however, low trauma distal radial fractures are not investigated well, by either Orthopaedic Surgeons or GP's. Fragility fractures are associated with underlying Osteoporosis & subsequent hip fracture and pharmacological treatments for Osteoporosis may reduce subsequent fracture by up to 50%. The correlation of Vertebral Wedge fractures and Osteoporosis is well understood by both specialities and these patients are more likely to be investigated.

The percentage of patients being investigated or treated for osteoporosis could be increased if Orthopaedic Surgeons highlighted this population to GPs or informed patients about the association with Osteoporosis. The role of Osteoporosis Nurse Specialist may prove beneficial in increasing the investigation Osteoporotic fractures and secondary prevention.

## Competing interests

The author(s) declare that they have no competing interest.

## Authors' contributions

All authors have made equal and substantial contributions to conception, design acquisition of data, analysis and interpretation of data, GC and LJ have been involved in drafting the manuscript. All authors read and approved the final manuscript to be published.

## Pre-publication history

The pre-publication history for this paper can be accessed here:



## References

[B1] (2000). Accidents, falls, fractures and osteoporosis: A strategy for primary care groups and local health groups.

[B2] Mallmin H, Ljunghall S (1994). Distal radius fracture is an early sign of general osteoporosis: bone mass measurements in a population-based study. Osteoporos Int.

[B3] Owen RA, Melton LJ, Ilstrup DM, Johnson KA, Riggs BL (1982). Colles' fracture and subsequent hip fracture risk. Clin Orthop Relat Res.

[B4] Finsen V, Benum P (1987). Colles' fracture as an indicator of increased risk of hip fracture. An epidemiological study. Ann Chir Gynaecol.

[B5] Melton LJ, Atkinson EJ, Cooper C, O'Fallon WM, Riggs BL (1999). Vertebral fractures predict subsequent fractures. Osteoporos Int.

[B6] Seeman E (1997). Osteoporosis: trials and tribulations. Am J Med.

[B7] Olmeda A, Greco F, Timar J, Malgaroli E (1995). Death rate in patients submitted to the surgical treatment of fracture of the proximal femur. Chir Organi Mov.

[B8] Dobbs MB, Buckwalter J, Saltzman C (1999). Osteoporosis: the increasing role of the orthopaedist. Iowa Orthop J.

[B9] Fithian DC, Page AE (1999). Osteoporosis prevention and the orthopaedic surgeon: when fracture care is not enough. J Bone Joint Surg Am.

[B10] Obrant KJ (1998). Prevention of osteoporotic fractures--should orthopedic surgeons care?. Acta Orthop Scand.

[B11] Pal B (1999). Questionnaire survey of advice given to patients with fractures. Bmj.

[B12] Skedros JG (2004). The orthopaedic surgeon's role in diagnosing and treating patients with osteoporotic fractures: standing discharge orders may be the solution for timely medical care. Osteoporos Int.

[B13] Majumdar SR, Rowe BH, Folk D, Johnson JA, Holroyd BH, Morrish DW, Maksymowych WP, Steiner IP, Harley CH, Wirzba BJ, Hanley DA, Blitz S, Russell AS (2004). A controlled trial to increase detection and treatment of osteoporosis in older patients with a wrist fracture. Ann Intern Med.

[B14] Pols HA, Felsenberg D, Hanley DA, Stepan J, Munoz-Torres M, Wilkin TJ, Qin-sheng G, Galich AM, Vandormael K, Yates AJ, Stych B (1999). Multinational, placebo-controlled, randomized trial of the effects of alendronate on bone density and fracture risk in postmenopausal women with low bone mass: results of the FOSIT study. Foxamax International Trial Study Group. Osteoporos Int.

[B15] McClung MR, Geusens P, Miller PD, Zippel H, Bensen WG, Roux C, Adami S, Fogelman I, Diamond T, Eastell R, Meunier PJ, Reginster JY (2001). Effect of risedronate on the risk of hip fracture in elderly women. Hip Intervention Program Study Group. N Engl J Med.

[B16] Meunier P (1996). Prevention of hip fractures by correcting calcium and vitamin D insufficiencies in elderly people. Scand J Rheumatol Suppl.

[B17] Shaw C (2000). 1998-based national population projections for the United Kingdom and constituent countries. Popul Trends.

